# Supplementary material for: Proteomic analysis of peripheral nerve myelin during murine aging
Source: Front Cell Neurosci. 2023 Oct 30;17:1214003. doi: 10.3389/fncel.2023.1214003 (PMC10642449; doi:10.3389/fncel.2023.1214003)
Supplement: Supplementary file 1 [file Data_Sheet_1.docx]

Supplementary Material

**Proteomic analysis of peripheral nerve myelin during murine aging**

**Dario Lucas Helbing^1, 2, 3, 4, 5^, Joanna M. Kirkpatrick^1^, Michael Reuter^1^, Julia Bischoff^1^, Amy Stockdale^1^, Annemarie Carlstedt^1^, Emilio Cirri^1^, Reinhard Bauer^5^ and Helen Morrison^1,6*^**

^1^ Leibniz Institute on Aging, Fritz Lipmann Institute, 07745 Jena, Germany

^2^  Department of Psychiatry and Psychotherapy, Jena University Hospital, Friedrich Schiller
 University Jena, 07745 Jena

^3^ Center for Intervention and Research on adaptive and maladaptive brain Circuits underlying
 mental health (C-I-R-C), Jena-Magdeburg-Halle

^4^ German Center for Mental Health (DZPG), Jena, Germany

^5^ Institute of Molecular Cell Biology, Jena University Hospital, Friedrich Schiller
 University Jena, 07745 Jena

^6^ Faculty of Biological Sciences, Friedrich-Schiller University, 07745 Jena, Germany

*** Correspondence:**Prof. Dr. Helen Morrison

Helen.Morrison@leibniz-fli.de

# Supplementary Figures

**Supplementary Figure S1**

**A** Venn diagram illustrating the number of myelin proteins detected in this study compared to that of Siems et al. (Siems et al., 2020). **B** Correlation plot of the mean Log_2_(iBAQ-values) of detected myelin proteins in young (left figure) or old nerves (right figure) to the mean PPM (= parts per million) values of the MS^e^-obtained myelin proteome dataset of young, wildtype mice from the Siems et al. study. The top 10 proteins with the highest mean iBAQ-values in each young or old myelin fraction, as well as the top 10 proteins for the Siems et al. myelin proteome data, are labelled with their corresponding gene names (top 10 young/old proteins in green, top 10 Siems et al. proteins in turquoise). Correlation statistics are shown in the upper left corner.


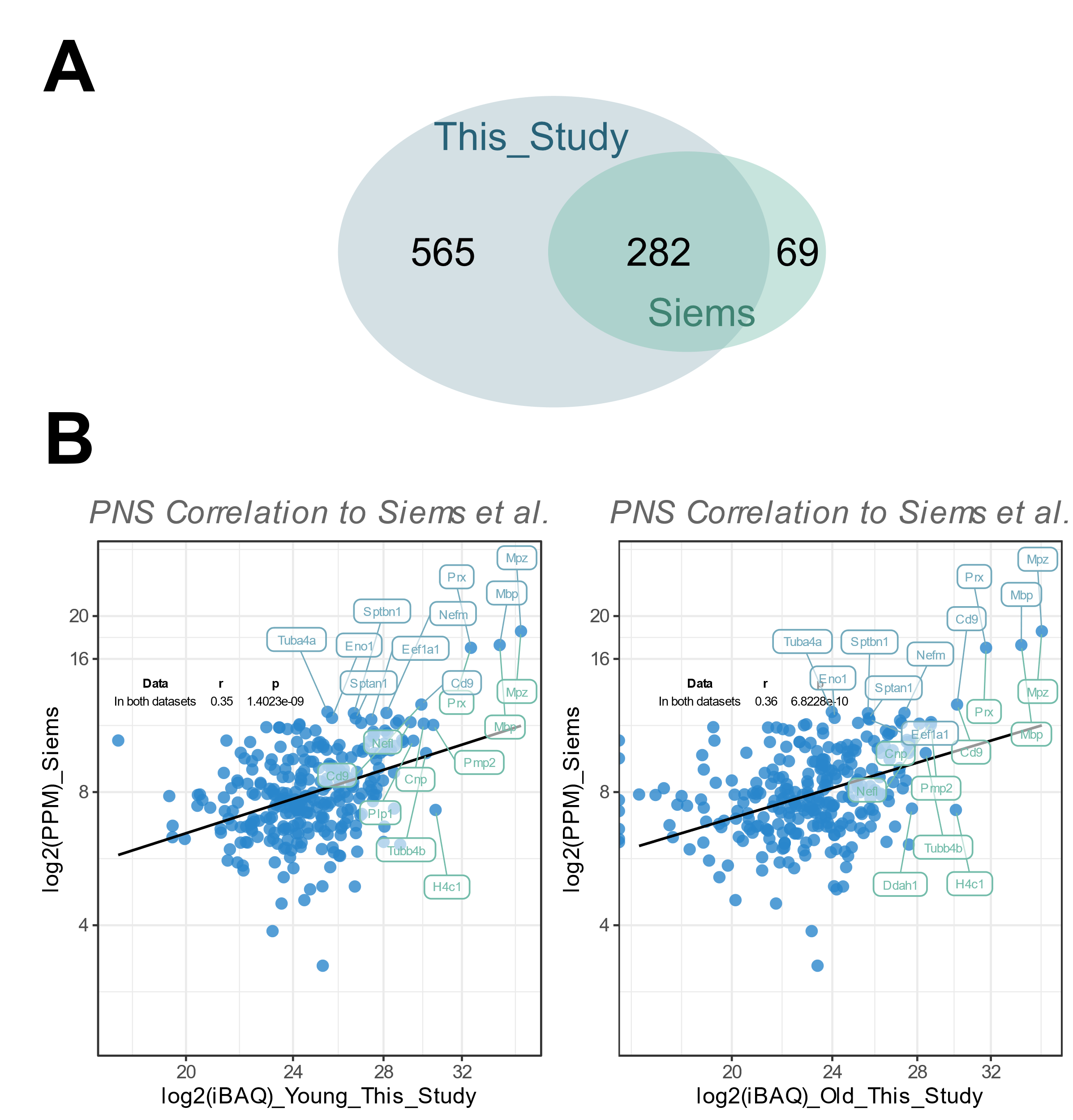


**Supplementary Figure S2**

Several marker proteins with a known pattern of regulation during peripheral nerve repair and regeneration were visualised with a heatmap. These include the repair Schwann cell marker proteins GAP43, c-JUN, p75NTR/NGFR and the macrophage marker protein Iba1/Aif1.


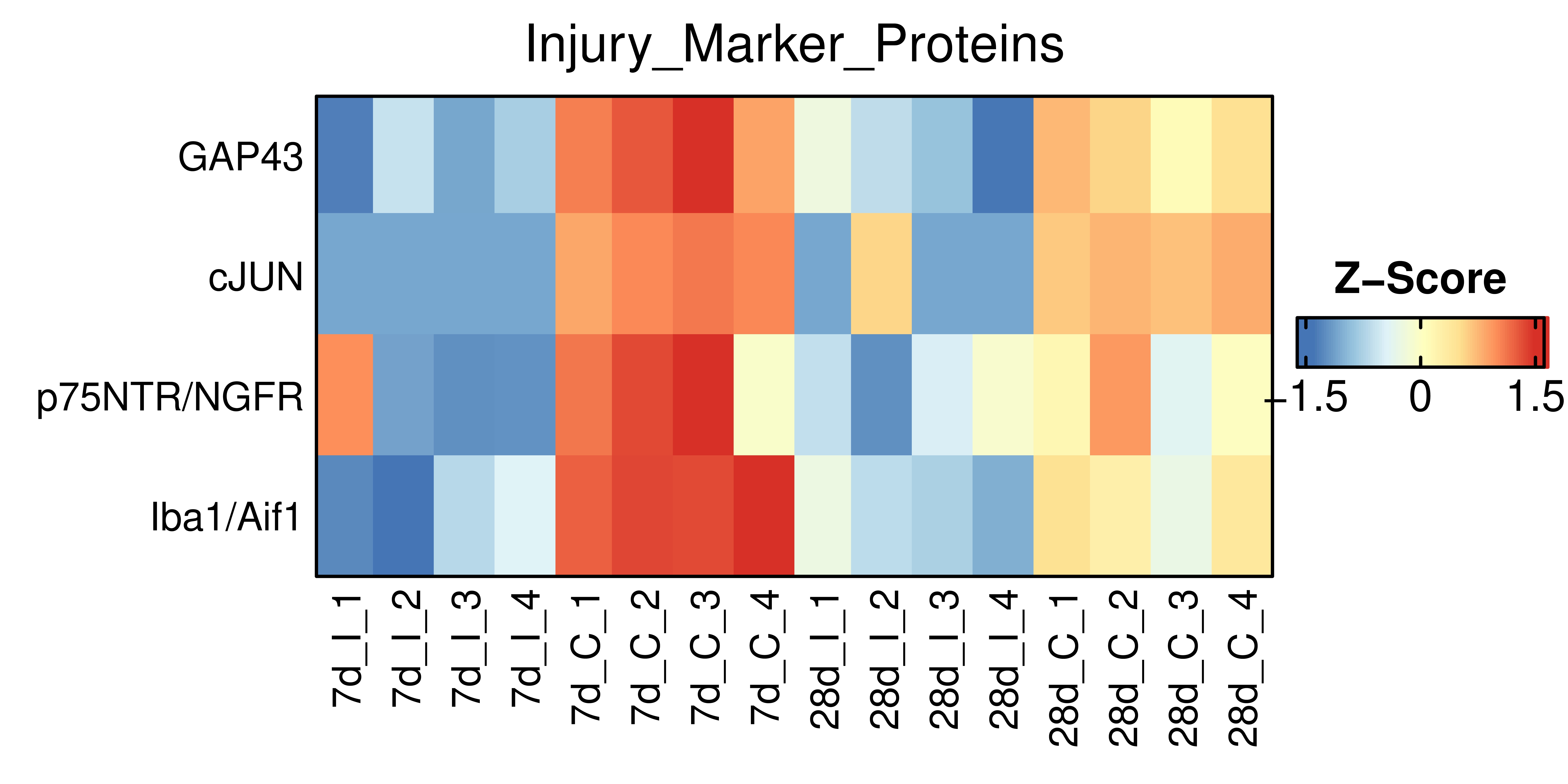


**Supplementary Figure S3**

**A** The myelin proteome obtained in this study was mapped to a proteomics dataset of peripheral nerve degeneration and regeneration, in which sciatic nerve proteomes were obtained at different timepoints post crush injury. “7d_I” refers to contralateral, intact control nerves from the same mice as the “7d_C” groups that refer to the crushed, ipsilateral nerves 7 dpc, n = 4 mice per group. Similarly, the “28d_I” and “28d_C” groups represent the intact, contralateral and crushed, ipsilateral nerves from the same mice in each case 28 dpc, n = 4 mice per group. Only the proteins are shown that were changed with a q-value < 0.05 in either the comparison 7d crush versus 7d intact (abbreviated as “A1”), or the comparison 28d crush versus 28d intact (abbreviated as “A2”). The protein-wise z-score normalized protein group intensities of all mapped myelin proteins are shown; on the right side of the heatmap an annotation of Log_2_ FC and q-value levels are depicted (│Log_2_ FC│≥ 0.58 in green or │Log_2_ FC│≤ 0.58 in orange, meaning more or less than 50% change and q-value < 0.05, respectively or a q-value > 0.05 in purple). **B** Bar charts of Log_10_(PG.Intensities) of all 7dpc or 28dpc differentially expressed myelin proteins are shown (n = 699 proteins). A Friedman-test was conducted, with a p-value ≤ 0.05. Post-hoc Dunn’s multiple comparison tests were conducted with * = p ≤ 0.05 for comparisons 7d crush versus intact and 28d crush versus intact and # = p ≤ 0.05 for the comparison 28d crush versus 7d crush.

**
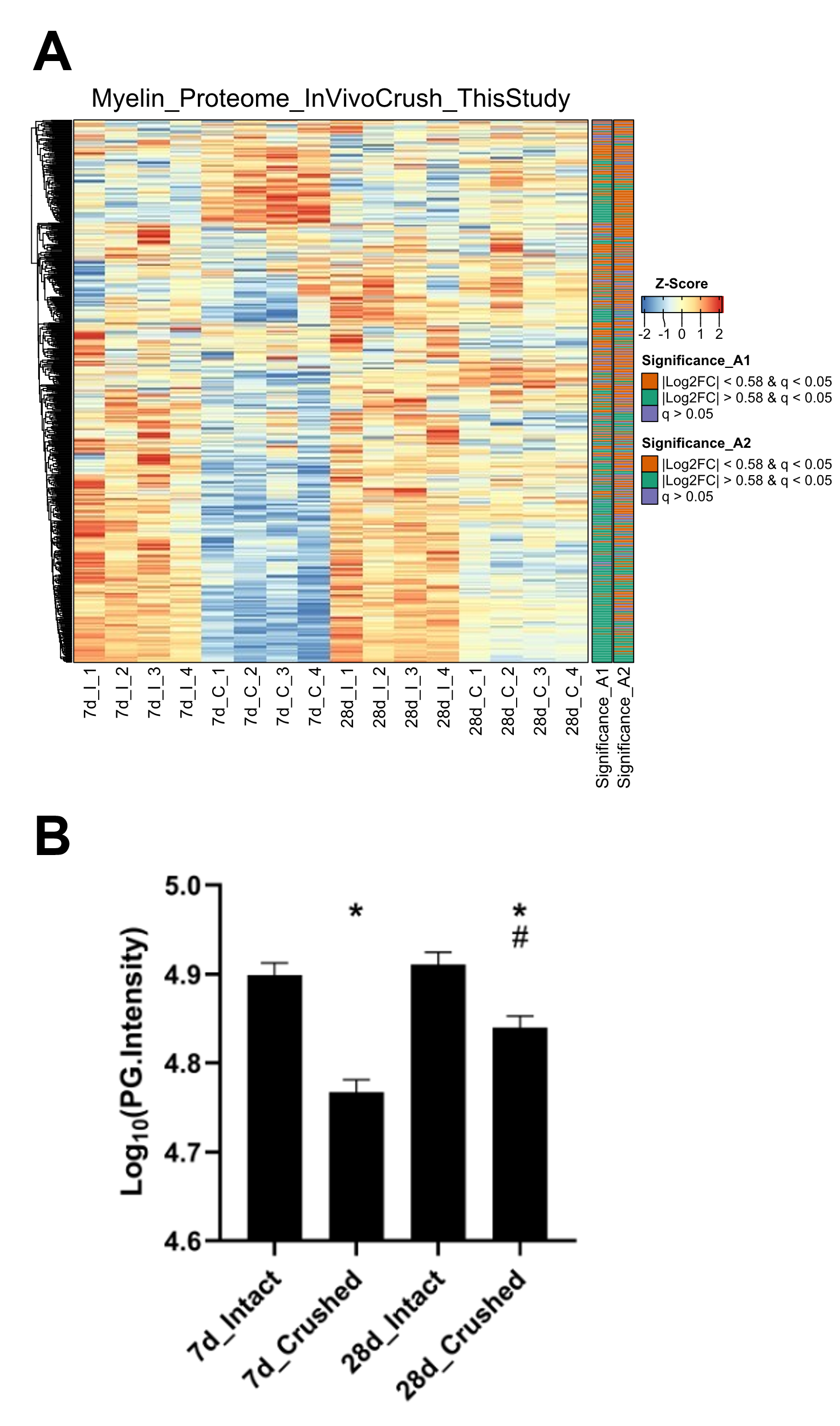
**

**Supplementary Figure S4**

**A** The myelin proteome determined by Siems et al. was mapped to the proteomics dataset of peripheral nerve degeneration and regeneration as shown in figure 2. Equivalently, “7d_I” refers to contralateral, intact control nerves from the same mice as the “7d_C” groups that refer to the crushed, ipsilateral nerves 7 dpc, n = 4 mice per group. The “28d_I” and “28d_C” groups represent the intact, contralateral and crushed, ipsilateral nerves from the same mice in each case 28 dpc, n = 4 mice per group. Only the proteins are shown that were changed with a q-value < 0.05 in either the comparison 7d crush versus 7d intact (abbreviated as “A1”), or the comparison 28d crush versus 28d intact (abbreviated as “A2”). The protein-wise z-score normalized protein group intensities of all mapped myelin proteins are shown; on the right side of the heatmap an annotation of Log_2_ FC and q-value levels are depicted (│Log_2_ FC│≥ 0.58 in green or │Log_2_ FC│≤ 0.58 in orange, meaning more or less than 50% change and q-value < 0.05, respectively or a q-value > 0.05 in purple). **B** Bar charts of Log_10_(PG.Intensities) of all 7dpc or 28dpc differentially expressed myelin proteins from the Siems et al. dataset are shown (n = 244 proteins). A Friedman-test was conducted, with a p-value ≤ 0.05. Post-hoc Dunn’s multiple comparison tests were conducted with * = p ≤ 0.05 for comparisons 7d crush versus intact and 28d crush versus intact and # = p ≤ 0.05 for the comparison 28d crush versus 7d crush.


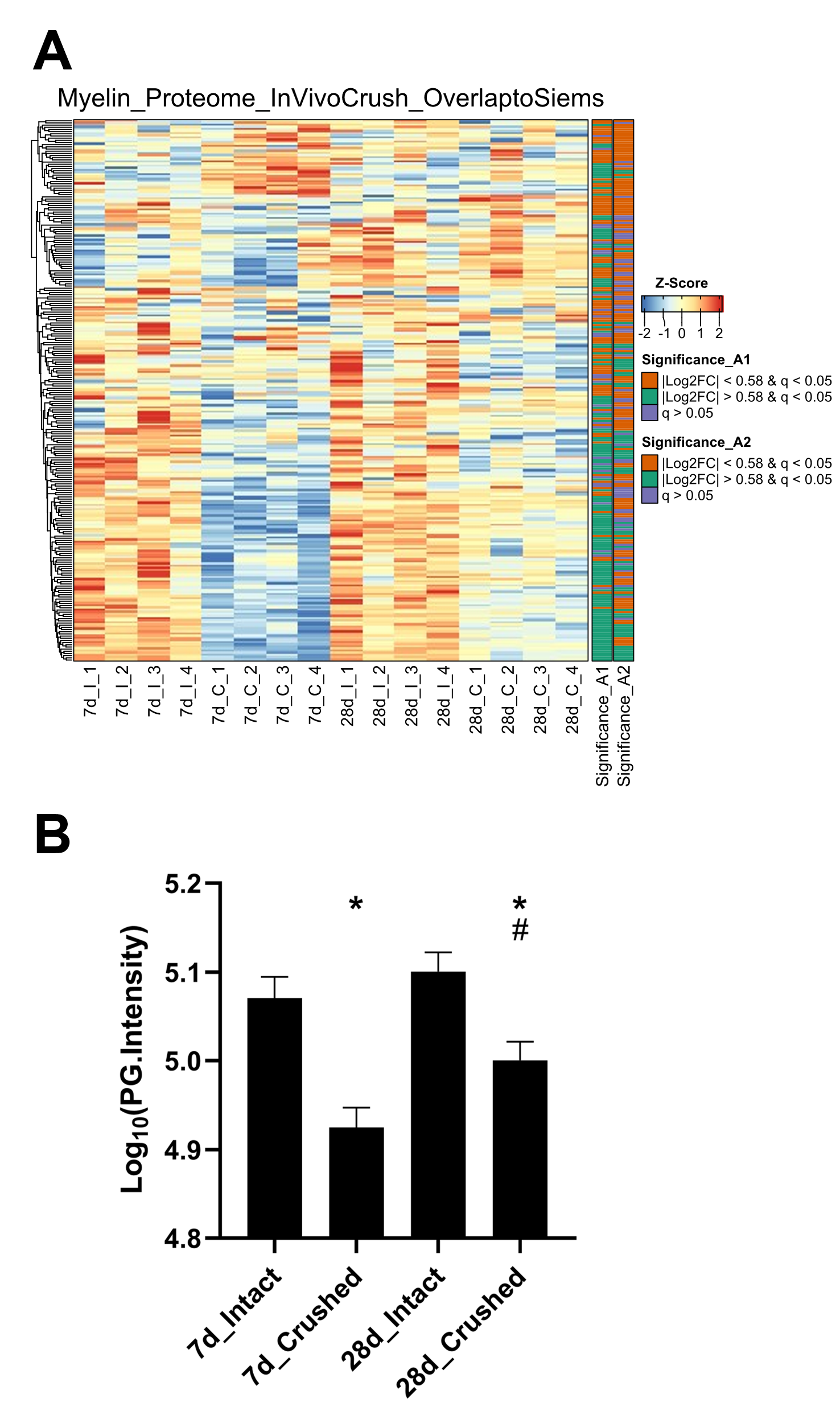


**Supplementary Figure S5**


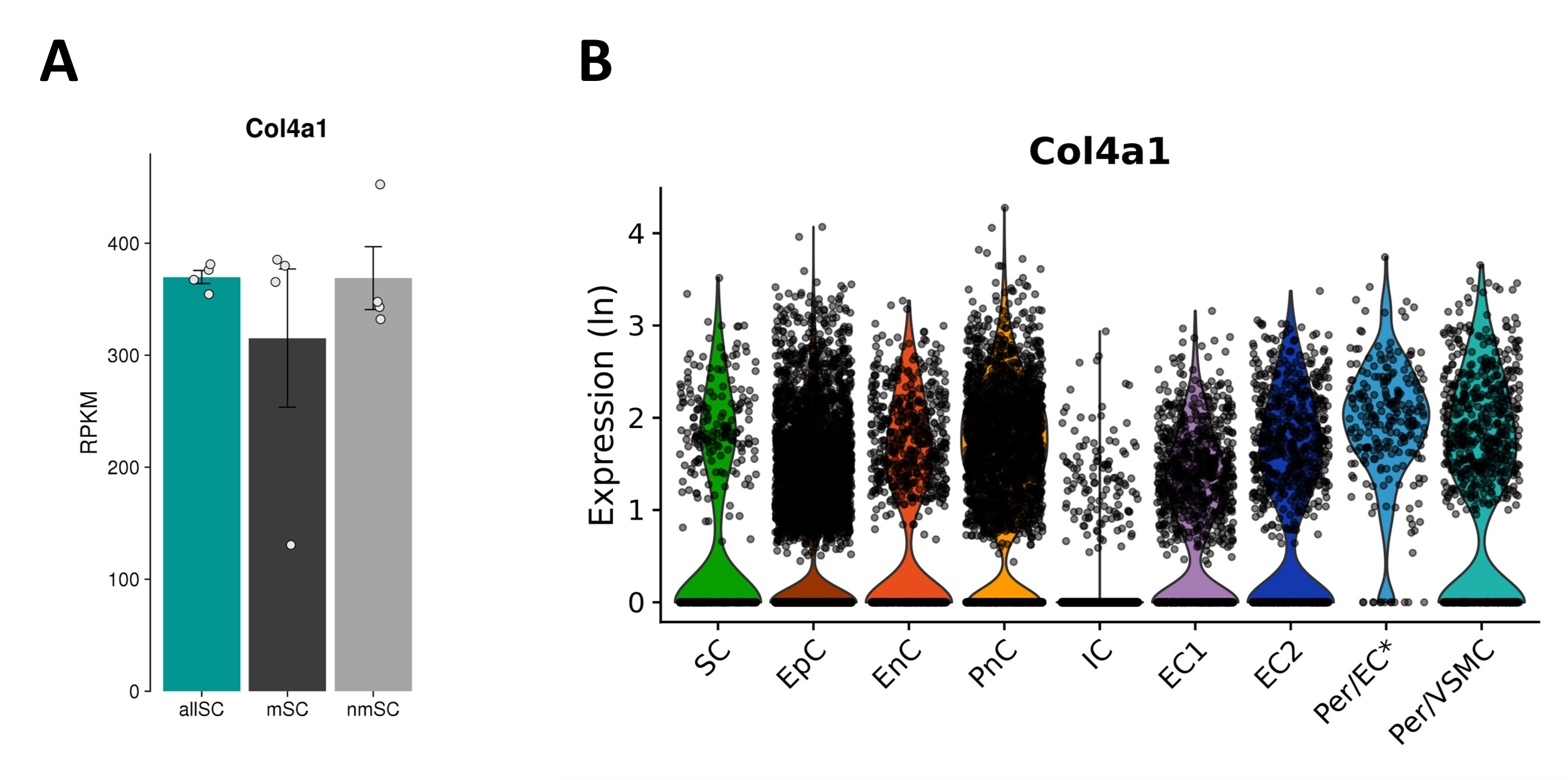
Re-analysis of the sciatic nerve Schwann cell bulk RNAseq (**A**) and whole sciatic nerve scRNAseq (**B**) datasets by Gerber et al. Data were obtained by searching for “Col4a1” on the website of the “Sciatic Nerve Atlas (SNAT)”, hosted by the authors of the original study at “https://snat.ethz.ch/” (Gerber et al., 2021) **A** Col4a1 expression in all (allSC), myelinating (mSC) and non-myelinating Schwann cells (nmSC). **B** Col4a1 expression levels across all classified peripheral nerve cell types at P60.

**Supplementary Figure S6**

Re-analysis of a scRNAseq dataset of intact whole sciatic nerves by Zhao et al. Data were obtained by searching for “Col4a1” on the website of the “Injured Sciatic Nerve Atlas (iSNAT)”, hosted by the authors of the original study at “https://cdb-rshiny.med.umich.edu/Giger_iSNAT/” (Zhao et al., 2022) **A** Overview UMAP plot showing all detected/classified cell types in intact mouse sciatic nerves **B** UMAP plot showing the scaled Col4a1 levels on a colour scale, with higher numbers indicating higher transcript levels.


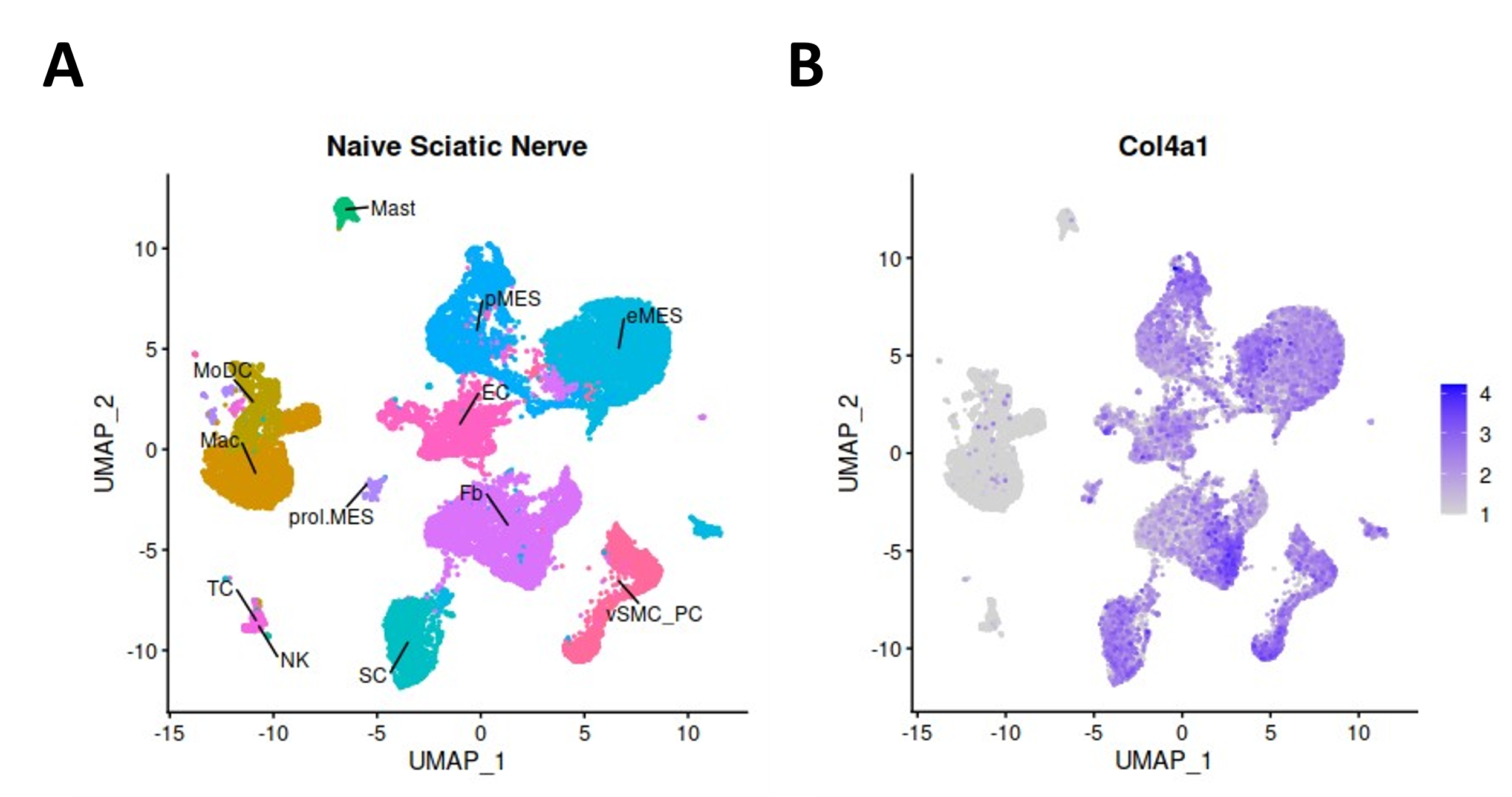


Gerber, D., Pereira, J.A., Gerber, J., Tan, G., Dimitrieva, S., Yanguez, E., et al. (2021). Transcriptional profiling of mouse peripheral nerves to the single-cell level to build a sciatic nerve ATlas (SNAT). *Elife* 10. doi: 10.7554/eLife.58591.

Siems, S.B., Jahn, O., Eichel, M.A., Kannaiyan, N., Wu, L.M.N., Sherman, D.L., et al. (2020). Proteome profile of peripheral myelin in healthy mice and in a neuropathy model. *Elife* 9. doi: 10.7554/eLife.51406.

Zhao, X.F., Huffman, L.D., Hafner, H., Athaiya, M., Finneran, M.C., Kalinski, A.L., et al. (2022). The injured sciatic nerve atlas (iSNAT), insights into the cellular and molecular basis of neural tissue degeneration and regeneration. *Elife* 11. doi: 10.7554/eLife.80881.
